# Positive suggestions via headphones during general anesthesia for the improvement of vegetative & cognitive postoperative course parameters in elderly orthopedic patients – a randomized controlled double-blinded trial (POSSUDEL)

**DOI:** 10.1186/s13063-025-09108-x

**Published:** 2025-10-27

**Authors:** Lena Heiß, Saskia Neitzert, Ernil Hansen, Hartmuth Nowak, Renate Neitzert, Thomas Saller

**Affiliations:** 1 Department of Anaesthesiology, LMU Hospital, Munich, Germany; 2 Department of Anaesthesiology, UKR Hospital, Regensburg, Germany; 3 Department of Anesthesiology, Intensive Care Medicine and Pain Therapy, Center for Artificial Intelligence, Medical Informatics and Data Science, Ruhr University Bochum, Knappschaft Kliniken University Hospital Bochum, Bochum, Germany

**Keywords:** Anesthesia, Processed EEG, Elderly, Headphones, Nociception level index, Pain, PONV, Positive suggestions, Delirium, Total intravenous anesthesia

## Abstract

**Background:**

Postoperative delirium and cognitive deficits are significant surgical complications, especially in elderly patients. The reported incidence of postoperative delirium is variable but notably high in cardiothoracic, orthopedic, and general surgery. The etiology of postoperative delirium is known to be multifactorial, with prevention being the most effective strategy currently available. This study aims to explore the potential benefits of positive suggestions delivered via headphones during general anesthesia on the incidence of postoperative delirium and improving postoperative pain, nausea, and cognitive outcomes in elderly orthopedic patients.

**Methods:**

This randomized controlled double-blinded trial will involve patients aged 60 and above undergoing elective hip or knee surgery under general anesthesia. Participants will be randomized into three groups: a control group receiving no auditory intervention and two intervention groups receiving positive therapeutic suggestions via headphones from either a male or female speaker. The primary outcome is the incidence of postoperative delirium within 5 days after surgery that will be assessed by using the 4AT and 3DCAM. Secondary outcomes include pain intensity, which is measured intraoperative by nociception level index (NOL) and postoperative by NRS, medication consumption as well as postoperative nausea and vomiting. Data will be collected before, during, and after surgery as well as 3 months after surgery.

**Discussion:**

This study hypothesizes that positive auditory suggestions can reduce postoperative delirium incidence, lower pain intensity as well as pain medication use, and decrease postoperative nausea, vomiting incidence, and severity. Additionally, gender differences in response to male versus female voices will be explored. The findings could offer a non-invasive, cost-effective method to enhance postoperative recovery in elderly patients, potentially leading to changes in perioperative care practices.

**Trial registration:**

DRKS00030589 prospectively registered 25.10.2022.

## Administrative information

**Table:** 

Title {1}	Positive suggestions via headphones during general anesthesia for the improvement of vegetative & cognitive postoperative course parameters in elderly orthopedic patients – A randomized controlled double-blinded trial (POSSUDEL)
Trial registration {2a and 2b}	DRKS00030589 prospectively registered 25.10.2022 Positive Suggestionen mittels Tonträger während Allgemeinanästhesie zur Reduktion von Delir und der Verbesserung vegetativer und kognitiver Verlaufsparameter: Eine multizentrische, doppelblinde randomisierte Interventionsstudie (POSSUDEL)
Protocol version {3}	29.08.2022 V1.5
Funding {4}	This study is part of the LMU hospital's own funding program
Author details {5a}	Lena Heiß*, Saskia Neitzert*, Renate Neitzert, Ernil Hansen, Hartmuth Nowak, Thomas Saller * joint first authorship
Name and contact information for the trial sponsor {5b}	n/a This study is part of the LMU hospital's own funding program
Role of sponsor {5c}	n/a This study is part of the LMU hospital's own funding program

## Introduction

### Background and rationale {6a}

#### Postoperative syndromes with cognitive deficits

Postoperative delirium (POD) is an acute neuropsychiatric syndrome. It is characterized by transient disturbances in attention, awareness, and cognition following surgery [1]. It typically manifests within the first few days after an operation and is marked by fluctuating symptoms, including confusion, disorientation, an altered level of consciousness, and impaired memory. POD is more common in elderly patients and those having major surgery. The incidence of POD varies widely and depends on the age of the patient cohort studied and the type of surgical procedure [2]. The highest incidences are found after cardiothoracic procedures, orthopedic and general surgery [3]. While the exact etiology is best described as multifactorial and not fully understood to date [4], it is known to be associated with increased morbidity, prolonged hospital stay, an elevated risk of long-term cognitive decline as well as increased mortality [5]. For example, Moskowitz et al. demonstrated that for elective surgery, in patients 50 or more years of age, 5-year mortality was increased 7.35-fold after the occurrence of POD (95% CI, 1.49–36.18) [6].

Postoperative cognitive dysfunction (POCD) describes a measurable decline in cognitive domains such as memory, attention, and processing speed that occurs after surgery, particularly in older adults [7]. According to the recommendations of the Nomenclature Consensus Working Group [8], POCD refers to cognitive decline identified at least 30 days after surgery and persisting for up to 12 months postoperatively, with cognitive changes detected within the first 30 days classified as delayed neurocognitive recovery. Although POCD is often transient, resolving within weeks to months, it may persist in some cases for extended periods. Potential contributing factors include the effects of anesthesia, surgical stress, and perioperative complications [39].

Neurocognitive disorder (NCD), in contrast, refers to a more sustained decline in cognitive abilities that is severe enough to interfere with everyday functioning.

As the development of POD or long-term POCD represents the most frequent postoperative complication, mainly affecting elderly patients [9], a further increase of this complication is expected in the future due to demographic change [6]. As prevention has proven most effectively [3], patient’s personal risk factors and possible predictors must be identified and measures to reduce the probability of the occurrence of POD/POCD or NCD [10] must be taken.

#### Importance of therapeutic communication

Communication plays a special role in the entire field of healthcare. It can accompany and support medical therapies. Appropriate use of language and professional conversation management allow us to discover patients’ resources and use them for the process of healing.

A randomized, double-blind, multicenter trial showed that audio cues can reduce pain and nausea, consequently reducing the use of analgesics and antiemetics in at-risk groups. The time to reorientation was also shown to be diminished [11]. Due to the age structure of the study, delirium incidence was inherently low, so no conclusions could be drawn regarding the antidelirogenic effect of positive suggestions.

#### Objectified intraoperative pain measurement

Measuring pain intensity is usually based on self-reporting by patients. With this method the subjective level of pain can be determined before and after surgery. Since patients cannot be questioned during general anesthesia, it was not possible to measure pain level intraoperatively in the past. Lately, an AI-based algorithm has been developed to generate a Nociception Level Index (NOL) during surgery [12]. The algorithm considers variants of the following four parameters, which can be measured via a single finger electrode: Skin conductance, heart rate variability, accelerometry, and skin temperature. Studies have already shown that the NOL index is suitable for predicting postoperative pain [13]. Thus, this technology seems to be suitable for a more precise analysis of the effect of therapeutic interventions especially on perioperative pain perception.

#### Gender differences: effect of different voices

Gender acts as an independent risk factor for several diseases and disorders. For example, female gender has already been established as a specific risk factor for the occurrence of postoperative nausea and vomiting [14]. The opposite seems to be true for the occurrence of delirium after surgical interventions: In this context, men seem to be more likely to develop POD [15]. With the upcoming awareness for gender sensitive medicine, research has also increasingly focused on the impact of sex differences on the efficacy of therapeutic interventions. For instance, there appear to be discrepancies in the efficacy of hypnosis therapy for smoking cessation when administered to different sexes (Green et al., 2008). However, robust data on gender differences in the effects of suggestive procedures is missing. In particular, no research has been done on gender differences in the effects of hypnotic suggestion when delivered by different voices.

#### Biomarkers to predict cognitive outcome after surgery

NCD, particularly POD and POCD represent significant complications following surgical procedures. Given that current therapeutic approaches primarily focus on general factors such as, e.g., fluid and electrolyte balance [16], there is a growing emphasis on early diagnosis and prevention of NCD [5]. The identification of high-risk patients in advance is of the utmost importance. For this purpose, biomarkers have been investigated [17]; however, their suitability for routine use is limited due to the invasive nature of the puncture. An alternative approach is to utilize markers in the blood, which can be categorized into three groups: global non-specific markers, established specific markers of neurocognitive functions and markers with less evidence to date. While so far, no single marker on its own has been sufficient for accurate prediction, a targeted combination of different markers could be an effective method for predicting cognitive complications with sufficient sensitivity and specificity [18]. As part of the ethically approved study protocol, perioperative blood samples were collected for the assessment of exploratory biomarkers potentially associated with postoperative cognitive and functional outcomes, such as cortisol, cortisol derivatives, NT-proCNP, NfL, S-100, pTau, and IL-6. These samples will be analyzed in a separate, hypothesis-generating investigation and are not part of the analyses reported in the present manuscript.

This trial will examine whether suggestions can reduce the probability of occurrence of delirium. Furthermore, it will be examined to what extent intraoperative application of different suggestive statements can reduce the postoperative perceived nausea as well as vomiting and pain, measured by the consumption of analgesics at a defined pain level. Moreover, it will be tested whether patients return to spontaneous breathing more rapidly, regain consciousness faster, are oriented and have less anxiety by listening to a sound recording with suggestions compared to a control group.

Potential risk markers for stress, delirium, and inflammation are to be investigated by measuring several parameters in the patients’ blood.

Beside the above mentioned targets, it will be investigated whether the choice of voice (male vs. female) has an influence on the effects described above.

### Objectives {7}

In the current study, we aim to address the following hypotheses:

Primary hypothesis:

1. Delirium incidence is lower in the intervention group than in the control group

Secondary hypotheses:

2) Pain intensity is lower in the intervention group than in the control group
3) Intraoperative: NOL values are lower in the intervention group than in the control group
4) Postoperative: NRS values are lower in the intervention group than in the control.
5) Pain medication consumption p.p. is lower in the intervention group than in the control group
6) Postoperative nausea and vomiting (PONV) incidence is lower in the intervention group than in the control group

Exploratory analyzation:

7) The above-mentioned effects differ depending on whether the subjects hear a speaker’s voice according to their own gender or not

### Trial design {8}

**Fig. 1:**
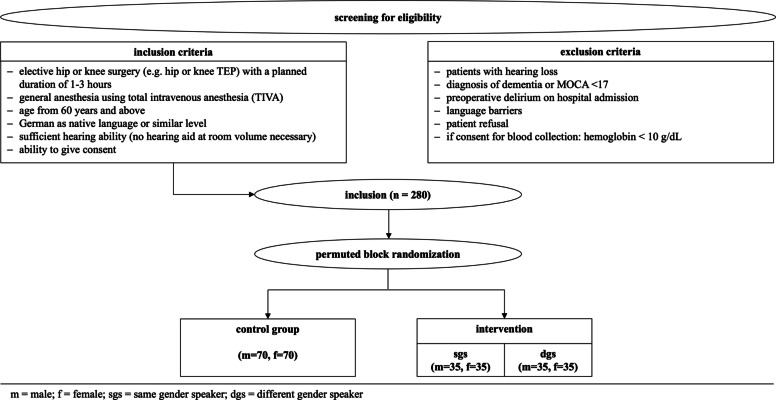
Flow chart

A parallel group design will be applied. The study physician, who is not directly involved in data collection, will randomly assign patients to experimental conditions using software. Every person directly involved will be blinded to the allocation, as will the patients. Included patients will be randomized into 3 groups. In the sense of a balanced design, equal numbers of patients will be assigned to the intervention or control group. The experimental group will be further subdivided. During general anesthesia, patients in both intervention groups will be presented with positive therapeutic content via headphones either from a female or male speaker. Patients in the control group also will receive headphones for general anesthesia, but the track that will be played does not contain any audio content. Otherwise, the treatment will not differ from the verum group. A flow chart of the process of recruiting, including and randomizing patient is shown in Fig. 1.

## Methods: participants, interventions and outcomes

### Study setting {9}

All data will be collected in Germany. Leading center of the trial is the hospital of the Munich Ludwig-Maximilians-University (LMU). Further data is planned to be collected in the hospital of the Ruhr University Bochum as well as in the Heidelberg university hospital.

### Eligibility criteria {10}

Patients meeting following criteria will be included:

- Elective hip or knee surgery (e.g., hip or knee TEP) with a planned duration of 1–3 h
- General anesthesia using total intravenous anesthesia (TIVA)*
- Age from 60 years and above
- German as native language or similar level
- Sufficient hearing ability (no hearing aid at room volume necessary)
- Ability to give consent

The following exclusion criteria apply:

- Patients with hearing loss
- Diagnosis of dementia or MOCA < 17
- Preoperative delirium on hospital admission
- Language barriers
- Patient refusal
- If consent for blood collection: hemoglobin < 10 g/dL

### Who will take informed consent? {26a}

Prior to surgery, patients who will undergo knee or hip surgery will be seen by a member of the study team who will ask them for their informed consent. If consent will be given, a short assessment will be conducted to evaluate inclusion and exclusion criteria.

### Additional consent provisions for collection and use of participant data and biological specimens {26b}

N/a (patient data will be specifically used for this study).

## Interventions

### Explanation for the choice of comparators {6b}

Delirium is a complication that primarily affects patients of older age. Therefore, patients will be recruited who already reached the age of 60 for all three subgroups. The control group will also receive headphones but without the suggestion audio.

#### Intervention description {11a}

Patients will be randomized with permuted block randomization that stratifies for their gender into a control and a intervention group within a 1:1 ratio. Intervention group will be further randomized 1:1 into a group that listens to audio content spoken by a voice that matches their gender and into a group that listens to a gender incongruent voice. The audio intervention is standardized and developed according to principles of therapeutic suggestion. We used the original text from Nowak et al. [11] as a blueprint for our intervention but adjusted it slightly to match our intentions. With the help of a soundcheck track volume will be set to a comfortable but clearly audible level before induction, with adjustments if necessary, prior to loss of consciousness. The audio content is structured into two segments. The initial segment begins after the intubation of the patient is being completed and therefore after loss of consciousness. It runs continuously (repeatedly) until approximately 15 min before the initiation of the extubation stage. The following text extracts are drawn from the first audio track (translated from the original German suggestion):

Positive suggestions to prevent delirium

*You are now sleeping soundly and deeply and can relax and rest, recover and recharge your batteries, because you are safe and well protected. Everything you hear, see, and feel contributes to your well-being. This allows your body to focus entirely on its self-healing powers. And we are by your side. You hear my voice, it accompanies you, you can concentrate fully on it, because what I tell you is important for you, important for your well-being and your healing.*

Positive suggestions to prevent pain:

*You will feel carefree and unaffected in the hours and days following the operation. You will feel calm, warm, relaxed, and content. Everything you feel will be good for your well-being. It will show you that your body is working hard to repair itself, restore balance, and return everything to normal. Your recovery can progress, unstoppable. Further and further.*

Positive suggestions to prevent PONV:

*Gradually, all your bodily functions will start up again: your circulation will get going, as will your digestion. Saliva will be produced and you will be able to swallow it. And you will be able to drink. Everything will start up again and move in the right direction, namely from top to bottom, from your mouth to your stomach and further and further into your intestines, moving steadily straight ahead. A sense of well-being can spread more and more and you can ask yourself what you feel like eating first. You will then be able to send blood flow back down to the intestines, which have been resting until then. And with all the energy and oxygen, they start working again. You can swallow liquid again And it flows down the esophagus into the stomach And from the stomach it is transported further into the intestines and in the intestines further and further, evenly and unstoppably in one direction: further*

Approximately 15 min before the initiation of the extubation stage, the audio track is being changed: Patients in the intervention group are now exposed to content that is specifically related to the process of waking up and the initial postoperative period. The following (translated from German) text extracts are drawn from the second audio track:

Positive suggestions for initiating wake up period:

*The operation went well and will be completed shortly. The healing process has already begun. You have been able to rest and regain your strength. Now it is time to come back to the here and now. All the fears that you wanted to protect yourself from are no longer necessary, because you are safe and well cared for.*

Positive suggestions to prevent pain:

*All pain that is actually intended to warn you of something is unnecessary, because you will receive every support.*

Positive suggestions to prevent PONV:

*Any nervousness that causes your stomach and digestion to slow down is unnecessary, because everything is running smoothly and normally. You are safe. Your stomach and intestines are getting back on track. Everything is moving in the right direction. You can swallow freely to clear your throat of saliva. You can look forward to drinking and eating. What will be the first thing you feel like eating?*

Positive suggestions to prevent delirium:

*Now shift your attention back from the inside to the outside. See the light, hear the sounds, you become more and more alert and start moving again. You can orient yourself and find your way around. Everything is fine.*

As soon as the propofol application is being stopped to wake up the patient, headphones will be removed. This is considered the end of our intervention. As the trial is double blinded, patients in the control group are presented with two different audio tracks as well, but these are empty tracks. For patients of all groups, there will be no background music withing the audio tracks.

Apart from the audio exposure, perioperative management will be identical in all groups: Anesthesia will be administered as total intravenous anesthesia (TIVA) using propofol for induction and maintenance, combined with sufentanil for intraoperative analgesia. Neuromuscular blockade will be achieved with rocuronium as needed. Depth of anesthesia will be monitored using bispectral index (BIS) monitoring. Standard multimodal analgesia, perioperative antibiotic prophylaxis, and postoperative nausea and vomiting (PONV) prophylaxis (dexamethasone and ondansetron, unless contraindicated) will be provided according to institutional protocols. No other aspects of anesthesia care will differ between study arms.

### Criteria for discontinuing or modifying allocated interventions {11b}

Consent to participate in the study can be withdrawn at any time, without giving reasons or obtaining any disadvantages for further medical care. In case of withdrawal from the study, personal data already obtained will be destroyed. Patients for whom there will be a short-term change in the anaesthetic procedure, e.g., to anaesthesia with sevoflurane, will be considered separately due to the increased risk of PONV.

### Strategies to improve adherence to interventions {11c}

N/a (as all patients will be monitored by a study team member during their surgery, no specific strategy to improve adherence to protocol is necessary).

### Relevant concomitant care permitted or prohibited during the trial {11d}

Participation in other delirium-related intervention studies.

### Provisions for post-trial care {30}

N/a (no special post-trial care needed).

### Outcomes {12}

Primary outcome will refer to delirium incidence. Cognitive testing in form of the 4AT [19, 20] as well as the 3DCAM [21] will be performed pre- and postoperatively on all study participants to determine whether the incidence of delirium is lower in the intervention group than in the control group.

The 4AT is a brief (< 2 min) screening tool for delirium and cognitive impairment. It includes four domains: alertness, abbreviated mental test–4, attention (months backwards), and acute change or fluctuating course. A score ≥ 4 suggests possible delirium. The 3D-CAM is a structured, validated instrument based on the Confusion Assessment Method (CAM) algorithm, optimized for administration in approximately 3 min. It evaluates acute onset or fluctuating course, inattention, disorganized thinking, and altered level of consciousness. A diagnosis of delirium according to 3D-CAM requires the presence of features 1 and 2, plus either feature 3 or 4 [21, 22]. Both assessments will be conducted by trained members of the study group. Correct assessment will be ensured by random checks as well as supervision through a psychologist.

As secondary outcomes postoperative nausea will be assessed with the Wengritzky PONV score [23], pain will be assessed using the Numerical Rating Scale (NRS) for subjective measurement and using the NOL index for objective measurement. Medication consumption will be documented by analyzing the patient’s files.

Furthermore, the effect of different speaker voices will be investigated as an exploratory analysis.

### Participant timeline {13}

The study duration for all included participants will be defined by the time of surgery until the day of discharge. A follow-up survey of the cognitive function level is planned after 3 months (follow up). The postoperative follow-up point in time was determined by the fact that, at this juncture, patients have customarily concluded their postoperative inpatient rehabilitation and have resided in their domestic environment for a period of several weeks. This period represents the earliest time frame in which daily functioning can be meaningfully assessed, without the confounding influence of ongoing rehabilitation measures or acute postoperative recovery processes. Therefore, it enables a valid evaluation of cognitive performance and its effect on daily living activities under stable conditions. Moreover, the timing of the follow-up assessment is consistent with international recommendations for the evaluation of postoperative POCD and associated outcomes. Evered et al. propose that cognitive testing for POCD should be conducted a minimum of 3 months after surgery, with the aim of avoiding the capture of transient, early postoperative changes [8]. While the MoCA was administered at baseline as an inclusion criterion to confirm adequate cognitive status at 3-month follow-up, no cognitive screening tool was applied. Instead, the assessment focused on functional outcomes using the SF-36 and iADL, as these measures are more directly aligned with capturing everyday functioning. Given the nationwide recruitment, follow-up instruments were selected to allow remote completion and ensure high response rates. Table 1 shows a detailed overview of the course of the study.

**Table 1:** Participant timeline

	STUDY PERIOD													
Date	Prior to surgery		Day of surgery						Day 1	Day 2	Day 3	Day 4	Day 5	+3 months
Timepoint	Screening	Baseline	Pre-OP	OP	PostOP recovery room				PostOP ward					Follow-Up
Visit-Code	SCR	V0	V1	V2	V3a	V3b	V3c	V3d	V4	V5	V6	V7	V8	V9
ENROLMENT														
Eligibility criteria	●													
Informed consent	●													
Feedback request	●													
ASSESSMENTS														
MOCA		●												
TMT		●												
Frailty Screening		●												
PONV risk-score		●												
Charlson Comorbidity Index		●												
Permanent medication		●												
QUESTIONNAIRES														
iADL		●												●
SF36		●												●
GDS		●												●
BDI														●
Complications														●
ALLOCATION														
Randomized allocation		●												
SAMPLES														
Blood sample			●											
Saliva sample			●											●
INTERVENTION														
Controll group				●										
SGS intervention group				●										
DGS intervention group				●										
OUTCOME VARIABLES														
4AT Delirium			●		●	●	●	●	●	●	●	●	●	
3DCAM Delirium								●	●	●	●	●	●	
Pain score (NAS)			●		●	●	●	●	●	●	●	●	●	●
Pain medication consumption			●					●	●	●	●	●	●	●
Comfort scale (NCS)			●		●	●	●	●	●	●	●	●	●	●
PONV wengritzky								●	●	●	●	●	●	
Sedline				●										
NOL				●										

### Sample size {14}

Based on the primary endpoint analysis, the sample size was calculated as follows:

At the university hospital, LMU Munich, a delirium incidence of approx. 25% can be assumed for electively orthopedic surgery. By means of therapeutic suggestion may be reduced by at least 50%. The calculation in G*Power [24] using the z-test “Difference between two independent proportions” results in a sample size of 110 subjects per group for a design in which the intervention group and control group are of equal size, with a required power of at least 0.8 and a significance level of 5%. This results in a total of 220 subjects.

Based on the results of the study by Nowak et al. 2020 [11], we also calculated the sample size needed to detect effects of the intervention on pain expression, which are of great interest in the secondary endpoint analysis: For effects on opioid use, an effect size of *d* = 0.36 is assumed. For a required power of 0.8 at a level of significance at 5%, a group size of at least 101 subjects is needed. For effects on pain intensity, an effect size of 0.325 can be calculated using G*Power based on the mean values and standard deviations given in the original work. When power is set at 0.8 and significance level at 5%, the resulting sample size amounts to a total of 248 subjects.

In addition to the effect of the intervention on delirium incidence, the effect on pain severity is also of great interest as a (secondary) endpoint. Hence, the larger calculated sample number (*n* = 248) is chosen as a further basis for calculation to be able to achieve sufficiently good power for the secondary endpoints as well.

To ensure that the calculated minimum power will be achieved, there is a need to take possible drop-outs into account. Therefore at least 10% more subjects will be necessary, which results in a total of 273 participants. In consideration of the balanced design, the final target equals in a rounded up total number of 280 patients.

## Recruitment {15}

At the LMU Munich university hospital, participants for the study will be recruited in the anesthesia outpatient clinic. If an eligible patient cannot be included in the anesthesia outpatient clinic, we will attempt to reach them through the orthopedics outpatient clinic.

### Assignment of interventions: allocation

#### Sequence generation {16a}

Included patients will be divided as follows: 1:1 (intervention vs. control group) and within the intervention group 1:1 to a speaker with the same gender (sGS) vs. a speaker with a different gender (dGS). Thus, the only factor influencing assignment is patient gender.

#### Concealment mechanism {16b}

Numbered audio players will be used to maintain blinding of the trained junior researchers conducting the intervention and data collection. After the randomization process, the study’s PI will notify the relevant parties regarding the designated number of the audio player to be utilized.

Those administering the intervention as well as the anesthetists in charge will not have permission to listen to the audio tracks on the different audio players.

#### Implementation {16c}

Trained junior researchers will enroll participants, while the allocation sequence will be automatically generated by RedCap. In detail, randomization will be conducted in REDCap using permuted block randomization with stratification by gender, resulting in separate randomization lists for male and female participants. This ensured a balanced allocation between intervention and control groups within each gender. The gender stratification was implemented to allow for the preplanned secondary analysis of potential effects related to gender–voice congruence. The PI of the study will perform randomization in the above mentioned way.

### Assignment of interventions: blinding

#### Who will be blinded {17a}

The trained junior researchers, the responsible clinical staff, and the patients themselves will be blinded to avoid any potential influence, whether conscious or unconscious.

#### Procedure for unblinding if needed {17b}

N/a (since our intervention will be supplementary and will not modify the process or medications, identification of blinding is unnecessary).

### Data collection and management

#### Plans for assessment and collection of outcomes {18a}

##### Baseline

Patients will be pseudonymized after inclusion. Required data items encompass Montral Cognitive Assessment (MOCA) [25], Frailty-test [26], PONV risk score [23], and continuous medication including pain medication. Additionally, patients will complete the Quality-of-Life questionnaire (SF-36) [27], Geriatric Depression Scale (GDS) [28], and instrumental Activities of Daily Living (iADL) [29].

##### Before surgery

Directly prior to their scheduled surgery patients will be visited for assessment. The patients’ cognitive state will be assessed using the 4AT. Additionally, they will be asked to rate their current level of pain using the NRS, as well as provide a saliva sample for cortisol analysis. Blood samples for cortisol levels and biomarkers will be obtained within the placement of a peripheral venous access, without the need for a separate puncture.

##### Throughout surgery

The following data will be recorded routinely. During anaesthesia depth of anaesthesia will be monitored intraoperative processed EEG, employing devices such as Sedline® [30]. Other crucial parameters are analgesic use, NOL-index [12] evaluated pain levels, overall operating time, and recovery duration, measured from the end of sedation to the time of extubating.

##### After surgery

After extubating objective data will be collected in the recovery room every 15 min for a total duration of 1 h. Postoperative assessments will include the 4AT score for delirium, pain score measured on NRS, Numerical Comfort Scale (NCS) for assessing comfort, and postoperative nausea rated from 0 to 3. After the patient will have been in the recovery room for 1 h, additional assessments will be conducted, including an extended delirium screening (3DCAM), PONV Score antiemetic consumption, and nurse-controlled analgesia consumption for NRS 3.

In the regular ward, data will be recorded for the first 5 days post-surgery: pain scores (measured using the NRS); analgesic intake via nurse-controlled analgesia (NCA; applicable for NRS of 3 or higher); PONV scores (as per Wengritzky’s classification system); antiemetic usage, comfort ratings (using the NCS); and delirium assessments (using both the 4AT score and 3DCAM) will be documented.

Follow-up assessments will be conducted 3 months after the surgery by sending patients a set of questionnaires (iADL, GDS, BDI, SF36) via mail. They will also be asked to provide their pain and comfort levels (NAS and NCS, respectively) and to submit another saliva sample. Any adverse effects that may have occurred will also be investigated and noted. If any cause for concern arises about returned questionnaires, the participants will be contacted and offered further assistance. All study participants may contact us at any time should they have any questions or concerns.

#### Plans to promote participant retention and complete follow-up {18b}

To enhance participant retention, trained junior investigators will supervise participants during their clinical stay. Study results as well as personal blood work results will be provided to patients at the study’s conclusion if they have agreed to receive them.

A 3-month follow-up will yield additional information on participants’ well-being, and, when needed, further supportive discussions will be arranged.

If participants choose to withdraw from the study, all data collected from them will be eliminated and excluded from the analysis. Withdrawal will be permitted at any point.

#### Data management {19}

The RedCap online tool (Vanderbilt University, Texas, USA) will serve as a shared database for all participating centers hosted by the Ruhr University Bochum. Patient data will exclusively be entered using pseudonyms, preventing any conclusions about the individuals without further information. During the study, data will be recorded on paper protocols or directly via RedCap. RedCap supports data quality assurance by enforcing specific data formatting and performing range checks during data entry. After completing data collection, we will conduct plausibility checks.

#### Confidentiality {27}

Patients’ confidential information will be protected under medical confidentiality and the German Federal Data Protection Act (BDSG). Pseudonymized proband data may be shared, but original documents will not be accessible to third parties.

#### Plans for collection, laboratory evaluation and storage of biological specimens for genetic or molecular analysis in this trial/future use {33}

On the day of surgery, a blood sample will be drawn to quantify relevant values and cryopreserve biomarkers. In addition, a saliva sample will be taken to analyze preoperative cortisol as a marker for experienced stress.

### Statistical methods

#### Statistical methods for primary and secondary outcomes {20a}

To ensure normal distribution, the Kolmogorov–Smirnov test will be conducted prior to further analyses. Statistical significance will be considered at a *p*-value < 0.05. The primary outcome (postoperative delirium incidence) and other binary outcomes (e.g., incidence of postoperative nausea and vomiting, PONV) will first be compared between the intervention and control groups using the chi^2^ test. Continuous non-parametric variables will be analyzed with Mann–Whitney *U* tests, and parametric variables with Student’s *t*-test. To account for potential confounding effects of intraoperative and postoperative medication use, detailed data on the type and dosage of analgesics and sedatives will be collected. These variables will be included as covariates in multivariable logistic regression models for binary outcomes (e.g., delirium, PONV) and multivariable linear regression models for continuous outcomes (e.g., postoperative pain scores, NRS; intraoperative NOL index values). Confounders will be selected a priori based on clinical relevance and evidence from previous literature to avoid data-driven overfitting.

Medication-related parameters (e.g., total opioid dose, intraoperative sedative use) will also be analysed descriptively and compared between groups. Sensitivity analyses will be performed to assess the robustness of the results with and without adjustment for these covariates.

#### Interim analyses {21b}

N/a (an interim evaluation is not intended).

#### Methods for additional analyses (e.g., subgroup analyses) {20b}

Gender effects will be explored by comparing the interventions group who listened to sGS with the intervention group who listened to dGS using the above-mentioned tests. In the same manner, we also aim to explore if there is a difference between female and male speaker voice in general.

#### Methods in analysis to handle protocol non-adherence and any statistical methods to handle missing data {20c}

In general, per-protocol analysis will be intended. To address the fact that complete protocol adherence is not always feasible in everyday clinical practice, all relevant cases will be included in the calculations as part of an intention-to-treat analysis.

#### Plans to give access to the full protocol, participant level-data and statistical code {31c}

N/a.

### Oversight and monitoring

#### Composition of the coordinating center and trial steering committee {5d}

N/a.

#### Composition of the data monitoring committee, its role and reporting structure {21a}

The study design, inclusion criteria, questionnaire selection, the points at which questioning takes place, together with supplementary monitoring and data-gathering monitors and the documentation tool RedCap were primarily prepared, selected and refined by the main center. In consultation with the other centers, details were negotiated through an iterative process to ensure a unified basis for conducting the study. As the study is self-funded and there are no sponsors involved, there are no competing interests or conflicts of interest. Trained junior researchers of the LMU Munich [27] hospital will monitor the process of data collection and report regularly to their head of trial.

#### Adverse event reporting and harms {22}

Adverse events, such as an increase in pain intensity or increased consumption of antiemetics, as well as delayed awakening after prolonged general anesthesia, will be closely monitored continuously and treated immediately.

#### Frequency and plans for auditing trial conduct {23}

Regular meetings will be held to review progress and suggest improvements and/or changes. As this will be a multicenter study, consultation and exchange with the other clinics will also be relevant.

#### Plans for communicating important protocol amendments to relevant parties (e.g., trial participants, ethical committees) {25}

No major changes are planned currently. If anything were to change, the ethics committee would be informed immediately so that the vote could be revised. As the trial is self-funded, there is no need to inform sponsors.

### Dissemination plans {31a}

The results will be published in a peer-reviewed medical journal, and the study participants will receive their personal laboratory results and the published article upon request and consent. The participating staff will be informed of the results of the study as part of an educational presentation.

## Discussion

The objective of our study is to examine the impact of positive suggestions on delirium in elderly patients, while also investigating the influence on postoperative pain and nausea.

Postoperative delirium (POD) remains a prevalent complication among elderly surgical patients, associated with increased morbidity and mortality [31]. The management of delirious patients poses substantial challenges for healthcare providers and families, often leading to significant financial burdens due to prolonged care requirements and potential long-term consequences [32]. Given the aging population and the rising number of surgeries performed on older adults, there is a critical need for effective preventive strategies and methods for early detection of delirium [6]. Such approaches have the potential to significantly improve clinical outcomes for this vulnerable population.

Evidence suggests that targeted interventions, such as positive suggestions, have a beneficial effect on postoperative symptoms such as pain perception, required pain medication and the incidence of postoperative nausea and vomiting (PONV) [11, 33]. It has also been shown that medical hypnosis has a favorable impact on individuals experiencing anxiety and stress [34].

Building upon these findings, this study aims to further investigate the impact of positive suggestions on delirium risk, postoperative pain, and PONV among patients over 60 years undergoing orthopedic procedures. Notably, this investigation incorporates an analysis of gender-specific differences and examines the influence of suggestion delivery by voices of different genders [35].

A major strength of this study design lies in its well-thought methodology, which was achieved through randomization, double-blinding and a multicenter approach. By specifically avoiding music in the control group and using spoken suggestions without background music in the intervention group, potential effects can be more directly attributed to the use of positive suggestions rather than being influenced by background music effects [36]. Additionally, to the best of our knowledge, this study is among the first within the field of delirium research to employ objective intraoperative pain assessment via the NOL index [12, 13]. This approach provides a more accurate and reliable measure of intraoperative pain, reducing the reliance on subjective patient self-reporting.

There are, however, several limitations that warrant consideration. Regular evaluation and delirium screenings throughout the study may themselves influence the neurocognitive state of patients. The necessary personal interactions and cognitive tasks involved in delirium assessments could be seen as a form of cognitive training, which is known to improve overall cognitive outcomes and may consequently affect delirium rates [37, 38]. Moreover, due to resource limitations, cultural and linguistic differences were not addressed, although they could offer valuable insights for future research.

In conclusion, the POSSUDEL study provides valuable insights into the potential of positive suggestions as a simple yet effective intervention to aid in the prevention of postoperative delirium. The findings may suggest clinically relevant strategies for enhancing treatment outcomes and general well-being in older patients, thus offering a practical tool for improving early detection and prophylaxis of POD in everyday clinical practice.

### Trial status

Enrollment began in November 2022. The study is still enrolling patients and is expected to complete data collection until October 2025.

## Data Availability

Only those directly involved in conducting the study will have access to the original final data of the trial. The data can be made available to the scientific community in anonymized form on reasonable request.

## Abbreviations

NRS: Numerical Rating Scale
PONV: Postoperative nausea and vomiting
SF36: Quality of Life Questionnaire
NCS: Numerical Comfort Scale
4AT: The 4 A’s Test: a rapid clinical test for delirium
3DCAM: 3-Minute diagnostic interview for CAM-defined delirium: a rapid clinical test for delirium
NOL: Nociception level (objectively quantify a patient’s physiological pain response during operation for example)
